# Supplementary material for: The interactome of intact mitochondria by cross-linking mass spectrometry provides evidence for coexisting respiratory supercomplexes
Source: Mol Cell Proteomics. 2017 Dec 8;17(2):216–32. doi: 10.1074/mcp.RA117.000470 (PMC5795388; doi:10.1074/mcp.RA117.000470)
Supplement: Supplemental Data [file supp_RA117.000470_133922_0_supp_23978_fzffwf.zip › spectra_annotation/mito_DR_spectra_annotation/326-2-10-1-7-1.pdf]

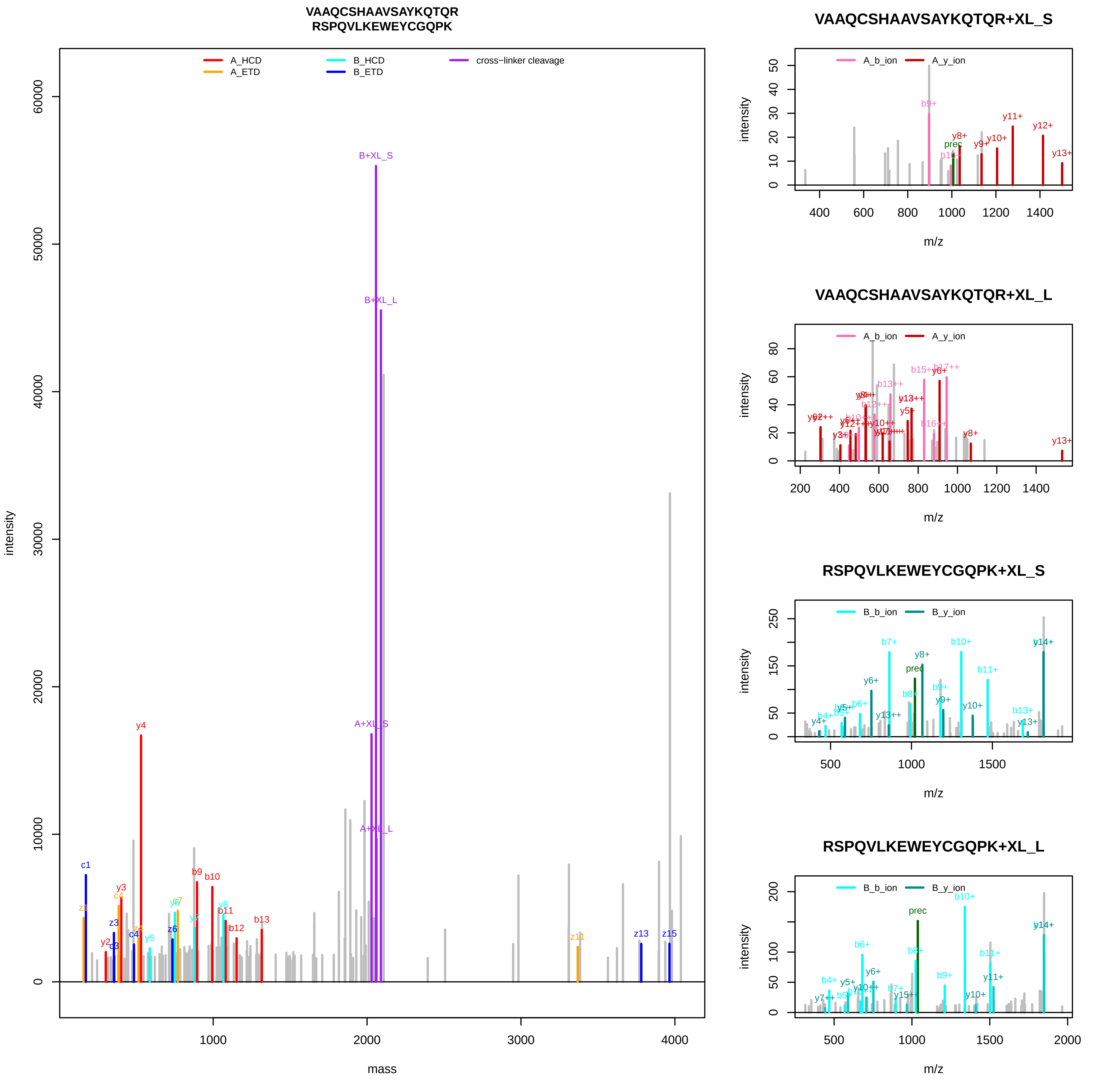

VAAQCSHAAVSAYKQTQR
RSPQVLKEWEYCGQPK
A_HCD
A_ETD
B_HCD
B_ETD
cross−linker cleavage
intensity
mass
0
10000
20000
30000
40000
50000
60000
1000
2000
3000
4000
B+XL_S
B+XL_L
A+XL_S
A+XL_L
y4
c1
z1
y2
z3
c3
y3
c4
z4
c4
y5
y6
c7
z6
y7
y8
b9
b10
b11
b12
b13
z11
z13
z15
VAAQCSHAAVSAYKQTQR+XL_S
VAAQCSHAAVSAYKQTQR+XL_L
RSPQVLKEWEYCGQPK+XL_S
RSPQVLKEWEYCGQPK+XL_L
A_b_ion
A_y_ion
B_b_ion
B_y_ion
m/z
prec
b9+
y8+
y9+
y10+
y11+
y12+
y13+
b15++
b17++
y6+
b13++
y13++
y8+
y5+
b7+
b10+
y8+
b11+
b9+
b8+
y9+
y10+
y6+
b6+
b13+
y13+
y14+
y11+
y15++
